# Demographics and Outcomes of Interhospital Transfer Patients Undergoing Intracranial Tumor Resection: A Retrospective Cohort Analysis

**DOI:** 10.7759/cureus.17868

**Published:** 2021-09-10

**Authors:** Ida Azizkhanian, Nicole Matluck, Jonathan V Ogulnick, Silvi Dore, Stergios Gatzofilas, Raeesa Habiba Hossain, Syed Faraz Kazim, Chad D Cole, Meic H Schmidt, Christian A Bowers

**Affiliations:** 1 School of Medicine, New York Medical College, Valhalla, USA; 2 Department of Neurosurgery, University of New Mexico Hospital, Albuquerque, USA

**Keywords:** interhospital transfer, cranial tumors, surgical outcomes, demographics, milan complexity scale, clavien-dindo score

## Abstract

Introduction

Interhospital transfer (IHT) contributes to increasing health care costs and typically accounts for increased patient morbidity and mortality compared to non-IHT patients. IHT inefficiencies leave patients vulnerable to delayed care and subsequent poor outcomes. In this study, we investigated factors influencing IHT of patients undergoing intracranial tumor resection (ITR), by comparing the variables distinguishing IHTs from non-IHT patients.

Methods

We performed a single-center retrospective review comparing IHT and non-IHT patients undergoing ITR from 2016 to 2018. Study variables included age, sex, race, the Milan Complexity Scale (MCS) score, 11-factor modified frailty index (mFI-11), length of stay (LOS), and Clavien-Dindo Score (CDS). Chi-square and Mann-Whitney U tests were used to identify significant differences in these variables between groups, while variables predictive of transfer status were identified using binary logistic regression.

Results

Data were collected from 219 patients undergoing ITR, with 80 (36.5%) IHT patients overall. The average age was 52 years (SD 18) and 57.7% were men. The MCS score was significantly higher in the IHT group (p = 0.014); however, mFI-11 was not (p = 0.322). The MCS score was predictive of IHT status in regression analysis (OR 1.17, p = 0.034). The IHT patients had a longer LOS (12 days vs 8 days, p = 0.014) with a lower CDS (p = 0.02).

Conclusion

The transfer patients for intracranial tumor resection had a higher MCS score and thus comprised a more surgically challenging population compared to non-transfer patients. As expected, IHT patients had a longer LOS as they lived further from hospital by definition.

## Introduction

Interhospital transfer (IHT) contributes to the burden of burgeoning health care costs as well as patient morbidity and mortality. This holds true emphatically for neurosurgical patients, for whom both avoidable IHT and inefficient transfer infrastructure leave room for quality improvement. In one series of neurosurgical patients, 57.4% of IHT led to no surgical intervention and in another one 34.5% IHTs were non-operative [1,2]. The direct transportation costs for avoidable transfers amounted to $1.46 million over two years in a study of neurosurgical IHT [2].

Inefficiencies in IHT infrastructure leave patients vulnerable to delayed care and poor outcomes. A previous study found that 17% of neurosurgical patients transferred to one hospital bypassed another neurosurgical center en route, and that in-transfer clinical decline occurred less frequently in patients who were transferred to the nearest neurosurgery center [3]. Furthermore, Holland et al. demonstrated that the majority of transfers at their institution occurred at night despite a near equal number of requests during the daytime [1]. They also found that weekend transfers tended to have poorer outcomes than weekday transfers [1]. Furthermore, a multicenter study on IHT found that the majority of patients with intracerebral hemorrhage who were transferred by helicopter did not even require emergent intervention upon arriving [4]. The same trends were also found in pediatric neurosurgery transfer patients, in whom most of the transfers occurred during evenings and weekends despite similar numbers of cases during the daytime and evening [5]. Particularly for pediatric neurosurgery IHTs, a single-institution study found that 42% of their transfers were unnecessary [6].

Furthermore, patient selection for IHT is unstandardized and vulnerable to bias and error. In a multicenter study using surveys from transferring physicians, receiving physicians, and patients in the ICU, the reasons rated as important to the transfer process varied across the three groups, and there was little to no agreement for specific reasons for the transfer [7]. Race may be other sources of bias in patient selection for IHT with white patients being more likely than people of other races to be transferred [2,7,8].

In the present study, we analysed intracranial tumor patients’ data who were transferred to our institution’s neurosurgery service, and investigated variables relating to disparities in transfer status and clinical outcomes.

## Materials and methods

We performed a retrospective cohort study, retrieving data from charts of adult patients who underwent surgery at our tertiary care center from 2016 to 2018 for intracranial tumor resection (ITR). Variables collected included patient age, race, insurance type, whether the patient was admitted through IHT or non-IHT, Milan Complexity Scale (MCS) score, patient frailty, hospital length of stay (LOS), and post-operative complications measured by Clavien-Dindo score (CDS).

Frailty is an age-independent reflection of physiological reserve that is frequently used in surgical outcome research [9-11]. To measure frailty, we used the 11-factor modified frailty index (mFI-11), an 11-point scale based on the Canadian Study of Health and Aging Frailty Index [12,13]. The mFI-11 predicts postoperative outcomes in many surgical subspecialties, including neurosurgery [14-16]. The mFI-11 was calculated retrospectively during chart review by tallying the comorbid conditions listed in the scale. The procedural complexity was defined by the MCS, outlined in Table 1.

**Table 1:** Milan Complexity Scale score

Feature	Score
Major brain vessel manipulation	1
Posterior fossa	1
Cranial nerve manipulation	2
Eloquent area	3
Tumor size > 4 cm	1
Total score	0-8

Categorical variables were analysed employing chi-squared goodness of fit test or Fisher’s exact test while non-parametric continuous variables were compared with the Mann-Whitney U test. Furthermore, the predictive value of variables was assessed using linear regression analysis or logistic regression for binary endpoints. All statistical analyses were performed using SPSS version 27.0 (IBM Corp, Armonk, NY) and Graph PadPrism version 9.0 (GraphPad Software, San Diego, CA) with the statistical significance set at p < 0.05.

## Results

The data were collected from total 218 patients: 80 (36.5%) IHT and 138 (63.5%) non-IHT patients. There was no significant difference in age, sex, mFI-11, race, or insurance type between the two groups (Table 2).

**Table 2:** Study cohort demographics and comparison between IHT and non-IHT groups MCS, Milan Complexity Scale; mFI-11, 11-factor modified frailty index; LOS, length of stay; CDS, Clavien-Dindo Score; IHT, interhospital transfer ^†^p < 0.05, statistically significant

Study variable	Cohort (n = 218)	IHT (n = 80)	Non-IHT (n = 138)	p-value
Age, yrs	52 (18)	52.9 (15.7)	51.3 (19.2)	0.684
M/F, n (%)	104 (47.7)/114 (52.3)	36 (45)/44 (55)	68 (49.2)/70 (50.7)	0.5424
Race, n (%)				
Caucasian	119 (54.6)	50 (62.5)	69 (50)	0.0015^†^
Hispanic	33 (15.1)	10 (12.5)	23 (16.7)	0.7963
Asian	6 (2.8)	1 (1.3)	5 (3.6)	0.3019
Black	37 (17)	11 (13.8)	26 (18.8)	0.3345
Other	24 (11)	8 (10)	15 (10.8)	0.8403
Insurance, n (%)				
Medicare	50 (22.9)	21 (26.3)	29 (21)	0.3755
Medicaid	46 (21.1)	13 (16.3)	33 (23.9)	0.1813
Private	98 (44.95)	32 (40)	66 (47.8)	0.2628
Uninsured	12 (5.5)	8 (10)	4 (29)	0.0267^†^
Other	12 (5.5)	6 (7.5)	6 (4.3)	0.8244
mFI-11	0 (1)	1 (1)	0 (1)	0.322
MCS	1 (3)	2 (3.5)	1 (3)	0.014^†^
CDS	1 (1)	1 (0)	1 (1)	0.02^†^
LOS (days)	9 (12.75)	12 (14.25)	8 (11.25)	0.014^†^

The MCS score was significantly higher in the IHT group (p = 0.014) and was predictive of IHT status in regression analysis (OR 1.17, 95% CI 1.012, 1.352, p = 0.034) (Table 3). The LOS was also significantly higher in IHT patients (Table 2); however, IHT was not predictive of LOS in regression analysis (OR 1.069, 95% CI -3.293, 11.092, p = 0.286).

**Table 3:** Study variable(s) predicting transfer status based on univariate logistic regression analysis MCS, Milan Complexity Scale ^†^Statistically significant

Study variable	OR (95% CI)	p-value
MCS	1.17 (1.012, 1.352)	0.034^†^

CDS was significantly lower in the IHT group (Table 2). In multivariate regression analysis, IHT status was predictive of CDS even when controlling for the MCS score (Table 4).

**Table 4:** Study variables predicting CDS based on multivariate regression analysis MCS, Milan Complexity Scale; CDS, Clavien-Dindo Score; IHT, interhospital transfer ^†^p < 0.05, statistically significant

Study variable	OR (95% CI)	p-value
MCS	0.019 (-0.65, 0.085)	0.785
IHT	-0.137 (-0.59, -0.005)	0.046^†^

## Discussion

We present a retrospective cohort study of 219 patients undergoing ITR to identify risk factors for IHT. Having increased surgical complexity was the only independent risk factor for IHT in this study. The MCS score was also predictive of IHT in regression analysis. While LOS was significantly higher in IHT patients, CDS was unexpectedly lower. IHT status was predictive of CDS in multivariate regression controlling for surgical complexity.

Our study contributes to the growing body of literature demonstrating worse outcomes in neurosurgical IHT patients, including higher LOS [2,17,18]. In a single-center study at a large academic center that included all neurosurgical patients over a two-year period, IHT patients had significantly longer LOS and ICU stay when compared to non-IHT patients [2]. Accordingly, IHT patients also had significantly higher treatment costs ($72,175 vs $46,163 for non-IHT patients) [2]. Our study adds to the previous work by confirming the relationship between IHT and LOS specifically in the subset of patients undergoing surgery for ITR.

MCS and IHT

To the best of our knowledge, this is the first study to investigate and quantify surgical complexity in the context of IHT in cranial tumors patients. Nonetheless, we have recently shown in spine patients that IHT patients comprise a surgically complex surgical spine population compared with non-IHT patients [19]. Here we found that IHT patients had significantly higher surgical complexity, as measured by the MCS, compared to non-IHT patients and that the increasing MCS is predictive of IHT status in binomial logistic regression. More surgically complex cases may be transferred to centers with surgeons who are presumably more familiar with difficult techniques. This, however, remains only an assumption as data from transferring and receiving hospitals has not been reported at these levels of detail. In support of this theory, Mueller et al. reported that hospitals with lower case mix index (CMI) were more likely to transfer out patients [8]. In the same nation-wide study using Medicare and Medicaid data, the investigators found that the variability in IHT rates between hospitals was not entirely accounted for by patient and hospital demographics [8]. More detailed record keeping of why transfers are initiated can help elucidate why variable practices exist between similar hospitals.

Pertinent negatives: decreased CDS in IHT

Several published studies support an association between IHT and higher mortality [1,2,20,21]. Contrarily, in the present study, we found that IHT patients had fewer complications as measured by CDS. Furthermore, MCS was not a predictor of CDS. In our cohort, there were only two mortalities, and therefore, a larger study would be needed to adequately investigate this endpoint. If CDS is lower in IHT patients, it begs the question of whether transferring these patients was necessary. If higher surgical complexity is an independent risk factor for being transferred but does not contribute to worse clinical outcomes, it should not be used as a factor for making decisions about IHT patient selection. Having adequate knowledge of what variables do or do not contribute to clinical outcomes for subsets of IHT patients will improve clinical decision making and reduce inefficiencies in the current practice. 

Limitations

In this study, we included only patients undergoing ITR to mitigate the confounding influence of data collected across surgical pathologies and from different providers. However, having collected data from a single center, our results may be more reflective of trends in IHT for ITR in our region or specific hospital network rather than those of the country in general.

It is clear from the literature that it is possible to have infrastructure in place that prevents wasteful transfers and worse morbidity and mortality among neurosurgical transfer patients. This is exemplified by a study from Albania that showed that implementing telemedicine reduced the incidence of unnecessary transfers [22]. Additionally, a large study from Tasmania, Australia, indicated that transfers to their tertiary referral center from peripheral hospitals were largely justifiable and that the patients did not fare worse than those who arrived at the tertiary center originally [23]. Needless to say, the factors that went into the success of these authors’ health systems in preventing wasteful and harmful transfers from occurring are complex, that are indeed attainable.

## Conclusions

Transfer patients (IHT) are a more surgically complex (MCS) population compared to non-IHT patients. Surgical complexity (MCS) was the only independent risk factor for IHT. While IHT status is associated with a longer LOS, a lower CDS was found in this group. Interestingly, patients’ insurance status, sex, race, and frailty did not significantly account for the variability in IHT.
